# Dose–response relationship between multiple trace elements and risk of all-cause mortality: a prospective cohort study

**DOI:** 10.3389/fnut.2023.1205537

**Published:** 2023-07-18

**Authors:** Shaohua Zhao, Shaohua Wang, Xiaorong Yang, Lin Shen

**Affiliations:** ^1^Department of Geriatric Medicine, Qilu Hospital of Shandong University, Jinan, China; ^2^Key Laboratory of Cardiovascular Proteomics of Shandong Province, Qilu Hospital of Shandong University, Jinan, China; ^3^Department of Internal Medicine, Jinan Hospital, Jinan, China; ^4^Clinical Epidemiology Unit, Qilu Hospital of Shandong University, Jinan, China; ^5^Clinical Research Center of Shandong University, Qilu Hospital, Cheeloo College of Medicine, Shandong University, Jinan, China

**Keywords:** trace element, mortality, dose–response, mixtures, epidemiology

## Abstract

**Objectives:**

We aimed to prospectively investigate the independent and combined relationship between trace elements concentrations [blood (selenium, manganese), serum (copper, zinc), and urine (cobalt, molybdenum, tin, strontium, iodine)] and all-cause mortality.

**Methods:**

This study included 5,412 individuals with demographical, examination, and laboratory data from the National Health and Nutrition Examination Survey. Three statistical models, including Cox proportional hazards models, restricted cubic spline models, and Bayesian kernel machine regression (BKMR) models, were conducted to estimate the longitudinal relationship between trace elements and all-cause mortality.

**Results:**

There were 356 deaths documented with a median follow-up time of 70 months. In the single-exposure model, the results showed that compared with the lowest quartile, the adjusted hazard ratios (HRs) of mortality for the highest quartile of selenium, manganese, and strontium were 0.47 (95% CI: 0.28–0.79), 1.57 (95% CI: 1.14–2.14), and 0.47 (95% CI: 0.26–0.86), respectively. A nonlinear relationship between zinc, cobalt and mortality was also observed. Furthermore, a significant overall effect of mixtures of trace elements on all-cause mortality was identified, especially when the mixture was at the 60th percentile or lower.

**Conclusion:**

The association of multiple trace elements with all-cause mortality was identified in this study. It is recommended that healthcare providers and relevant public health agencies should strengthen the surveillance and management of trace elements. Emphasis should be placed on monitoring the sources of trace elements such as the body, food, and environment. More population studies and animal experiments should be conducted to identify the underlying mechanisms.

## 1. Introduction

As an essential component, trace elements play an important role in maintaining the functions of the body. Trace elements can be ingested from food as well as enter the body from contaminated external environments. With increased industrialization, trace elements exposures in the environment have become a major source of impact on normal physiological functions in the body and have become one of the global public health challenges. Trace elements in the environment can enter the body through multiple routes, such as inhaling contaminated air (1), drinking contaminated water (2), and ingesting contaminated food (3, 4), as well as through dermal contact (5). At the same time, since some trace elements are extremely degradable and accumulate in the body, widespread low doses exposure in the general population may also cause serious health hazards.

Extensive epidemiological studies have shown that exposure to some trace elements may lead to numerous adverse health outcomes, including cardiovascular disease, neurological damage, kidney damage, and many cancers (6–10). For example, arsenic (As), cadmium (Cd), copper (Cu), nickel (Ni), and lead (Pb) may be associated with the development of diabetes mellitus (11–15). Other prospective studies have demonstrated that levels of Cd, Pb, As, antimony, and tungsten are associated with the risk of hypertension, peripheral, cardiovascular disease, and metabolic syndrome (16–20). Notably, these adverse events do not occur only after high levels of exposure; on the contrary, many harmful outcomes occur at exposure levels below safe exposure doses. Besides, previous studies mainly focused on the association between sole trace elements and specific diseases and also lacked population-specific findings (21, 22). Thus, exposure assessment and monitoring of low-dose trace elements in the general population, especially in vulnerable individuals, should be given widespread attention from the medical community both nationally and internationally.

The independent and combined relationship between trace elements and all-cause mortality has not been well established yet. Therefore, we performed a prospective cohort study to examine the independent and joint associations between multiple trace elements exposures and all-cause mortality by leveraging a large, nationwide database representative of the US population.

## 2. Materials and methods

### 2.1. Study design and population

The National Health and Nutrition Examination Survey (NHANES) is a series of surveys aimed at assessing the health and nutrition status of adults and children in the United States. The survey includes interviews questionnaires, laboratory testing analyses, and medical examination information. Interviews questionnaires included demographic, socioeconomic, and health-related questions. The examination component consists of medical, dental, and physiological measurements, as well as laboratory tests conducted by trained medical personnel. Meanwhile, the NHANES obtained accurate estimates representing the civilian population of the United States by using a complex multi-stage sampling survey and providing appropriate sample weights for this random subsample. Based on the above, this study will systematically explore the association between trace elements and all-cause mortality. The data source of the NHANES can be obtained on the Centers for Disease Control and Prevention National Center for Health Statistics website.^1^

**Figure 1:**
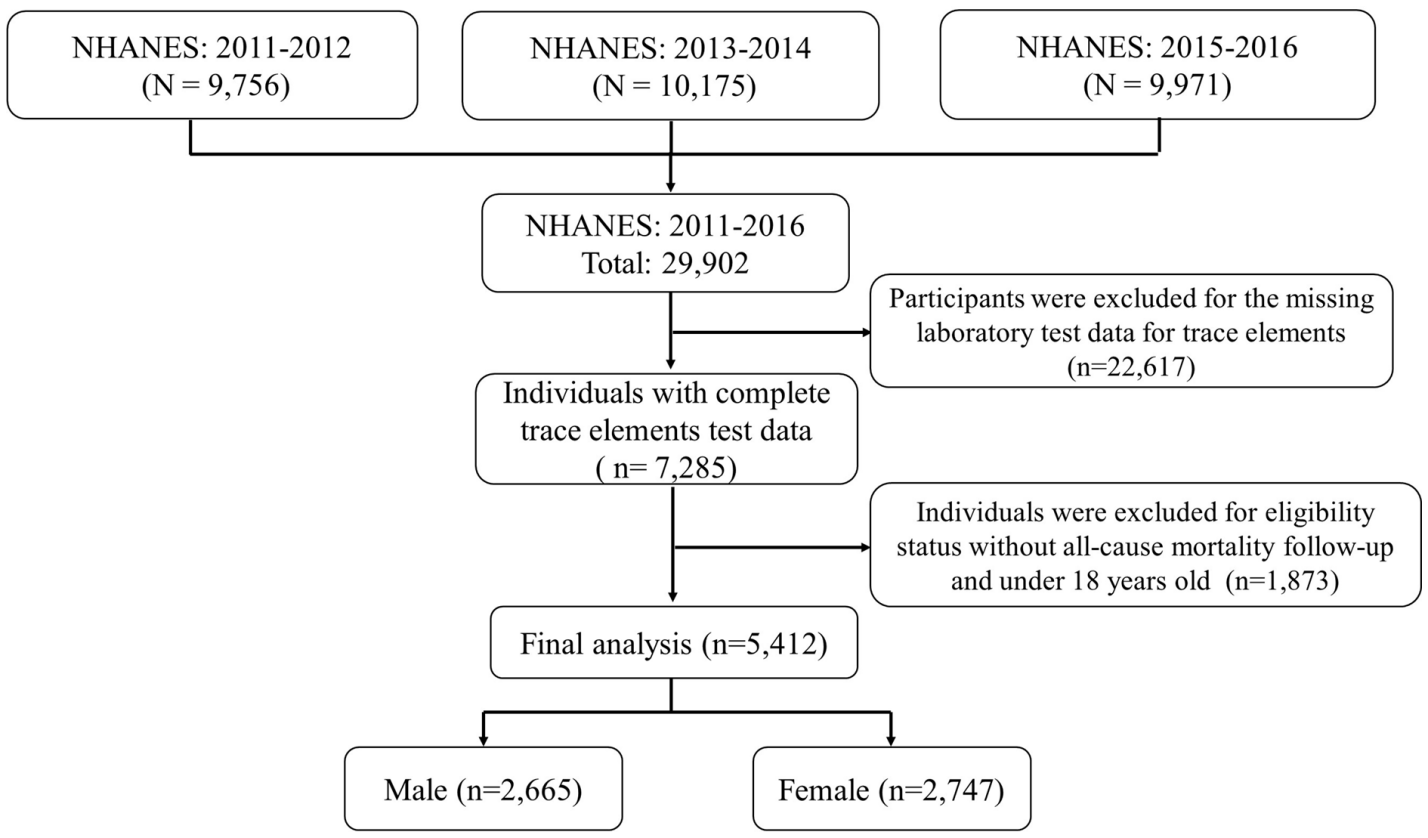
Eligible participants and those included in the analyses of the associations between trace elements and risk of all-cause mortality.

In this study, we studied and implemented data from 3 cycles of the NHANES from 2011 to 2016 (2011–2012, 2013–2014, and 2015–2016). All participants’ survey information was obtained by matching demographic databases and other data (including laboratory data for trace elements (blood, serum, and urine), examination data, laboratory data, and questionnaire data) through the use of the unique survey participant identifier (SEQN). Finally, a total of 5,412 individuals were enrolled in our study and the corresponding flow chart for selecting the study population is shown in Figure 1. Besides, the NHANES protocol was approved by the National Center for Health Statistics (NCHS) Institutional Review Committee, and all participants provided written informed consent.^2^

### 2.2. Measurement of trace elements levels

Our study included 9 trace elements {blood [selenium (Se), manganese (Mn)], serum [copper (Cu), zinc (Zn)], and urine [cobalt (Co), molybdenum (Mo), tin (Sn), strontium (Sr), iodine (I)]}, as it can give a more realistic reflection of the internal exposure dose of trace elements *in vivo*. Whole blood, serum, and urine specimens are directly measured using inductively coupled plasma mass spectrometry (ICP-MS, ELAN^®^ DRC II, PerkinElmer, Norwalk) after a simple dilution sample preparation procedure. ICP-MS is an analytical technique capable of performing trace element analysis. The diluted liquid sample enters the mass spectrometer through an inductively coupled plasma (ICP) ionization source and is atomized into small droplets. The small droplets in the aerosol are selectively passed through the spray chamber into the ICP by the flowing argon gas stream. The ions pass first through a focusing zone, then a dynamic reaction cell (DRC), a quadrupole mass filter, and finally a selective and rapid counting at the detector, allowing the individual isotopes of an element to be identified. Detailed descriptions regarding the specimen collection and processing procedures and the laboratory quality control are available at the NHANES website (see footnote 1). Meanwhile, according to NHANES guidelines, for analytes with analytical results below the lower limit of detection, results below the level of detection are assigned a value of LOD divided by the square root of 2 [LLOD/sqrt (2)]. We also provide more details on the limit of detection (LOD) for Co, Mo, Sr., Sn, Mn, Se, I, Zn, and Cu in Supplementary Table S1.

### 2.3. Determination of mortality status

To identify the mortality status of the follow-up population, we used the latest NHANES Public Use Link mortality file (LMF) as of December 31, 2019, which was correlated with the NCHS’s National Death Index (NDI) via an enhanced linkage algorithm. The public use LMF therein provides mortality follow-up data (including follow-up outcomes and timing) from the date of survey participation through December 31, 2019. The detailed linking methodology and analysis guidelines can be accessed on the NCHS Data Linking page.^3^

### 2.4. Covariates

Relevant information about social demographics, lifestyles, and health status is available through using individual and household demographic questionnaires during the NHANES household interviews. The covariate selection criteria were based on previously published studies and outcome-related variables available in the NHANES (23–25). Missing observations in covariates were removed for subsequent analysis. These variables mainly include demographic characteristics, physical measures, and behavioral information. The information is collected through standardized medical questionnaires in personal surveys. Demographic characteristics include age, sex (male, female), race/ethnicity (Mexican American, Hispanic, non-Hispanic white, non-Hispanic black, and other), marital status (married, unmarried), and education levels (less than 9th grade, 9−11th grade, high school grad/GED or equivalent, some college or associates degree, college graduate or above), family income-poverty ratio (PIR, <1. 0, ≥1.0 and ≤ 3. 0, >3. 0). Participants who reported smoking at least 100 cigarettes during their lifetime were considered smokers, and those who consumed at least 12 drinks in the last 12 months were considered alcohol users. Vigorous physical activity was defined as an activity that greatly increases the breathing or heart rate for at least 10 min continuously. Moderate physical activity was defined as an activity that causes small increases in breathing for at least 10 min continuously. Body mass index (BMI) is calculated by dividing weight (kg) by height (meter) squared, and our study classified all participants into three weight-status groups: normal (BMI of <25), over-weight (25 < BMI < 30), or obesity (BMI of ≥30) (23). More details of the questionnaire information can be accessed on the website.^4^

### 2.5. Statistical analysis

As the trace element data distribution was right skewed, the trace element concentrations in whole blood, urine, and serum were log-transformed to approximate a normal data distribution before subsequent statistical analysis. And extreme values in the data were also eliminated by winsorizing to reduce the effect of possible false outliers. Continuous variables are expressed in the form of the mean (standard deviation) and median (interquartile range, IQR), and categorical variables are expressed in terms of frequency and percentage. When comparing inter-group differences between the still-alive and deceased groups, the Chi-squared test was used for categorical variables and the Mann–Whitney *U* test or *t*-test for the continuous variables. Correlation heatmaps are used to recognize correlations between trace elements (Co, Mo, Sr., Sn, Mn, Se, I, Zn, and Cu).

We divided each trace element exposure level into four groups by quartiles (<25th, 25th ~50th, 50th ~75th, >75th). And Cox proportional risk regression models were performed using the quartiles of trace element exposure as independent variables, with the 25th quartile set as the reference group. The Schoenfeld residuals were used to assess the proportional hazards assumption and the results showed no significant deviation from proportionality over time. Dose–response relationship between each trace element exposure and mortality also was investigated using restricted cubic spline plots based on multivariate Cox regression. Restricted cubic splines allow for flexible modeling of non-linear associations by dividing the range of the exposure variable into multiple segments and fitting a cubic polynomial within each segment. Four knots (at percentiles 5, 35, 65, and 95% of the distribution) were established in our models, and P for nonlinearity was calculated. Three separate independent models were then built to calculate the adjusted hazard ratio (HR) and 95% confidence intervals (CIs) for mortality to avoid potential confounding factors. Model 1 is the unadjusted model. Model 2 adjusted for age and sex; based on model 2 variables, education levels, race/ethnicity, marital status, smoking status, alcohol consumption, PIR, BMI, and physical activity were added to model 3 for full model adjustment. Test for linear trends based on the medium value of each group as continuous variables.

Furthermore, given the potential for non-linear and non-additive dose–response relationships between mixed exposures on outcomes, Bayesian Kernel Machine Regression (BKMR), a unique semi-parametric modeling technique, was employed to assess the combined effects of mixed exposures on all-cause mortality risk. Briefly, the BKMR model can demonstrate how the individual exposure-outcome relationships change when fixing the levels of other mixtures at pre-specified levels, quantifying the relative contribution of each exposure using posterior probabilities of inclusion (PIP). The method was developed by integrating Bayesian and statistical learning methods to estimate interactions in non-linear and/or exposure-outcome associations. This uses a Markov chain Monte Carlo algorithm, which sets the number of iterations at 10,000. Following previous studies, potential interactions between specific exposure-response curves between elements were shown in our study when exposure levels of all other trace elements were maintained at the median or 25th, or 75th percentile. Adjustments for gender, age, race, education, PIR, marital status, smoking, alcohol consumption, BMI, and physical activity were performed in models. More relevant information about the BKMR models has been previously described in detail (26–28). The R program was used in all statistical analyses of our study (version 4.0.3),^5^ and ggplot, and RMS packages of the R program were used for results visualization. The *p*-value on both sides of less than 0.05 was considered statistically significant.

## 3. Results

### 3.1. Baseline characteristics of study participants

In this study, a total of 29,902 participants were surveyed. Of these, 22,617 were excluded because they did not participate in laboratory testing for trace elements. And 1,873 were excluded because of missing all-cause mortality data. Finally, left a total of 5,412 individuals available for our analysis, which included 2,665 males and 2,747 females (Figure 1). The average age of the final study population was 47.39 ± 18.35 years; 50.9% were female. By December 31, 2019, 356 participants were identified as deceased. The median follow-up time was 70 months (range 1–111 months). Baseline characteristics of the study population according to mortality status were presented in Table 1. Non-Hispanic White participants, College education, married, alcohol consumption, family PIR >1 and < 3, and never physical activity also accounted for a higher proportion of all participants.

**Table 1:** Baseline characteristics of the study individuals who were still alive vs. those who were deceased by December 31, 2019.

Variables	Still alive (*N* = 5,056)	Deceased (*N* = 356)	*p*-value^1^
Age (years), mean (SD)	45.89 ± 17.68	68.86 ± 13.86	<0.001^*^
Sex, n (%)			
Male	2,453 (49.0)	212 (60.0)	<0.001^*^
Female	2,603 (51.0)	144 (40.0)	
Race, n (%)			<0.001^*^
Mexican American	737 (15.0)	28 (7.9)	
Other Hispanic	571 (11.0)	21 (5.9)	
Non-Hispanic White	1,837 (36.0)	206 (58.0)	
Non-Hispanic Black	1,087 (21.0)	77 (22.0)	
Other Race	824 (16.0)	24 (6.7)	
Education level, n (%)			<0.001^*^
Less than 9th grade	466 (9.2)	64 (18.0)	
9-11th grade	571 (11.2)	64 (18.0)	
High school graduate	1,099 (21.7)	81 (23.0)	
College	1,553 (30.7)	87 (24.0)	
Graduate	1,367 (27.2)	60 (17.0)	
Marital status, n (%)			<0.001^*^
Married	3,003 (59.4)	179 (50.0)	
Unmarried	2,053 (40.6)	177 (50.0)	
BMI, n (%)			0.418
<25	1,561 (31.9)	121 (34.0)	
25–30	1,604 (31.7)	112 (31.5)	
>30	1,891 (37.4)	123 (34.5)	
PIR, n (%)			<0.001^*^
<1	1,066 (23.0)	80 (24.0)	
1–3	1,870 (41.0)	170 (52.0)	
>3	1,666 (36.0)	77 (24.0)	
Tobacco smoking, n (%)			<0.001^*^
Yes	2,050 (41.0)	219 (61.0)	
No	2,996 (59.0)	137 (39.0)	
Alcohol drinking, n (%)			0.356
Yes	3,455 (68.0)	246 (69.0)	
No	1,601 (32.0)	110 (31.0)	
Physical activity, n (%)			<0.001^*^
Never	3,045 (60.0)	271 (76.0)	
Moderate	1,044 (21.0)	58 (16.0)	
Vigorous	967 (19.0)	27 (7.3)	

*N* = 5,412. SD, standard deviation, boldface indicates significant associations (^*^p < 0.05).

^1^*p*-values were derived using Pearson chi-squared test for categorical variables and the Mann–Whitney U tests or *t*-test for continuous variables. PIR, Poverty-income ratio; BMI, body mass index.

**Figure 2:**
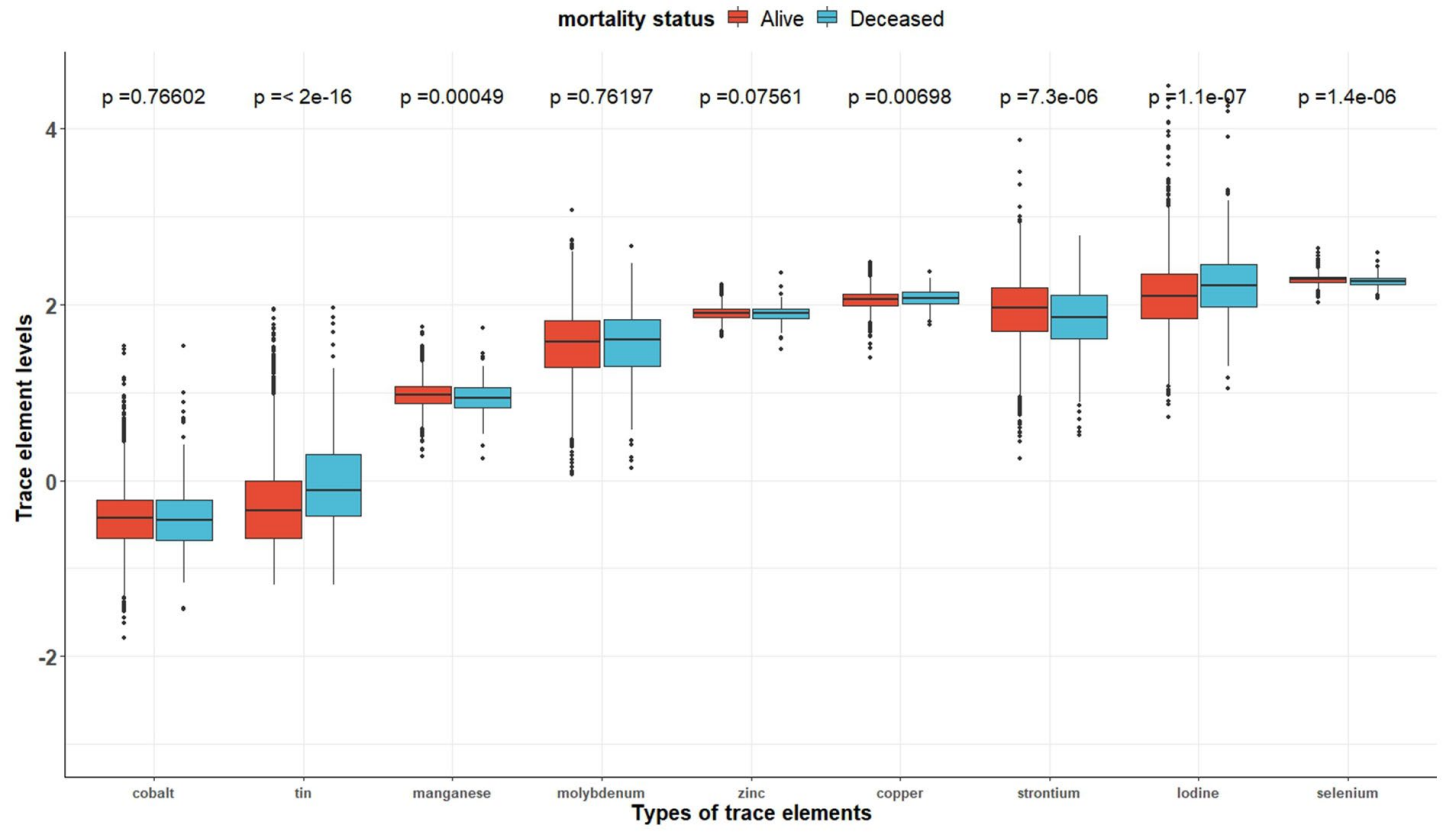
The distributions of trace element concentrations between two exposure subgroups (still alive vs. deceased).

Table 2 shows the distribution of the nine trace element concentrations between the two groups (the alive group and the deceased group). Figure 2 shows the visualization of the differences between the two groups. And Supplementary Figure S1 indicates that the correlation between the concentrations of the different trace elements (Co, Mo, Sr., Sn, Mn, Se, I, Zn, and Cu) is generally weak (cor < 0.4).

**Table 2:** Distribution of several trace elements in the study individuals.

Trace elements	Still alive (*N* = 5,056)	Deceased (*N* = 356)	*p*-value
Copper (μg/L), Median (IQR)	114.10 (98.50, 133.70)	119.30 (102.95, 137.55)	0.003^*^
Zinc (μg/L), Median (IQR)	80.40 (71.20, 90.40)	79.25 (68.38, 89.10)	0.048^*^
Selenium (μg/L), Median (IQR)	193.01 (179.08, 208.36)	185.47 (167.21, 203.29)	<0.001^*^
Manganese (μg/L), Median (IQR)	9.37 (7.56, 11.74)	8.77 (6.80, 11.37)	<0.001^*^
Cobalt (μg/L), Median (IQR)	0.37 (0.22, 0.60)	0.35 (0.21, 0.60)	0.739
Molybdenum (μg/L), Median (IQR)	38.17 (19.50, 66.40)	39.59 (19.70, 67.58)	0.794
Tin (μg/L), Median (IQR)	0.45 (0.22, 0.99)	0.77 (0.40, 2.02)	<0.001^*^
Strontium (μg/L), Median (IQR)	92.13 (49.96, 157.70)	72.64 (40.77, 127.99)	<0.001^*^
Iodine (ng/mL), Median (IQR)	125.10 (69.10, 225.40)	163.20 (95.90, 290.10)	<0.001^*^

Boldface indicates significant associations (^*^p < 0.05).

### 3.2. Associations between individual trace elements and the all-cause mortality risks

**Figure 3:**
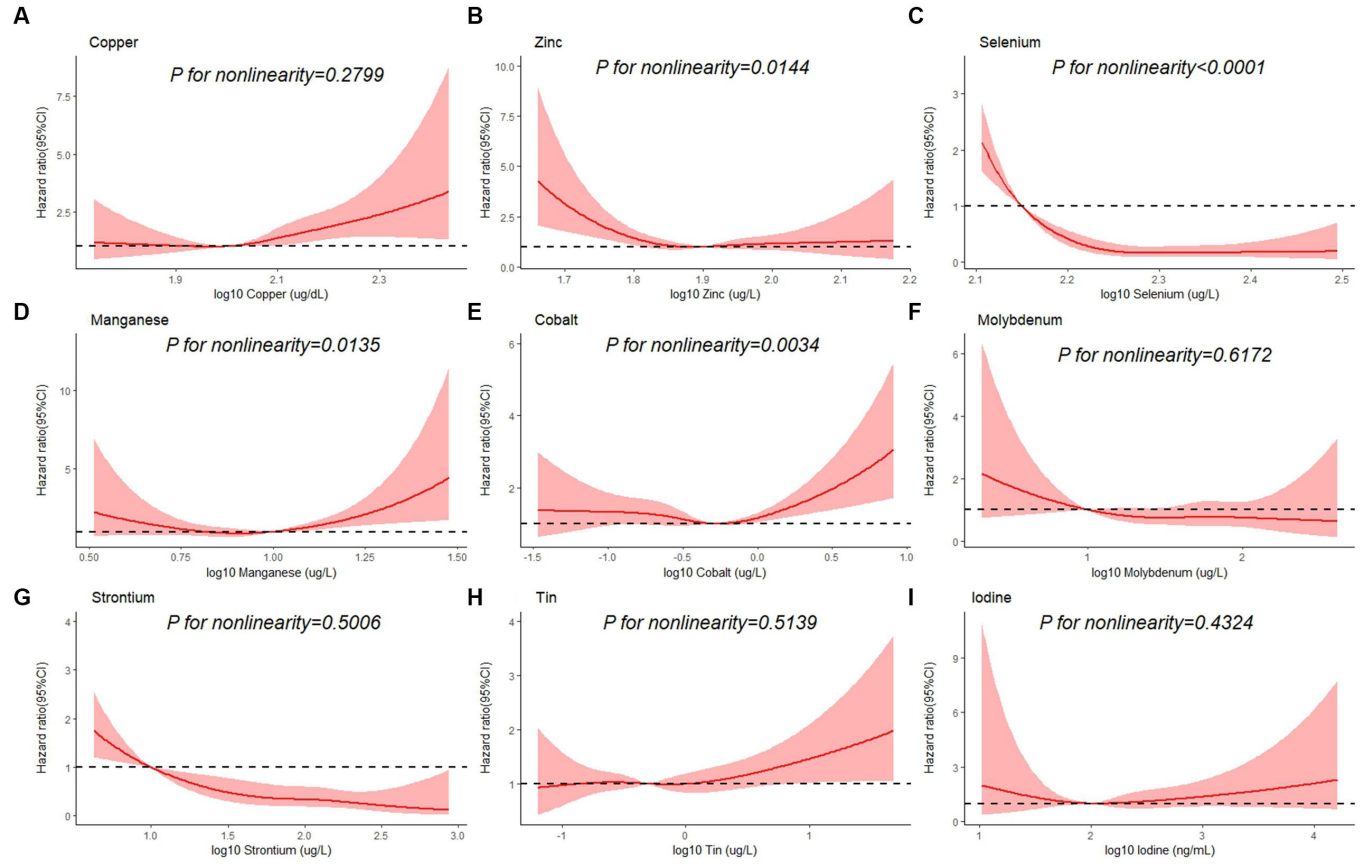
Restricted cubic spline plots of the association between all-cause mortality and log 10-transformed concentration of trace elements of **(A)** copper, **(B)** zinc, **(C)** selenium, **(D)** manganese, **(E)** cobalt, **(F)** molybdenum, **(G)** strontium, **(H)** tin, and **(I)** iodine. The associations were adjusted for age, sex, education level, race/ethnicity, marital status, tobacco smoking, alcohol drinking, BMI, PIR category, and physical activity.

Table 3 shows the association between each trace element and the risk of all-cause mortality by using the Cox proportional hazards model. After adjustment for full confounding factors, compared with the lowest level, the multivariate-adjusted HR and 95% CI for the highest quartiles were 1.61 (95% CI, 0.90–2.87), 0.81 (95% CI, 0.59–1.12), 0.47 (95% CI: 0.28–0.79), 1.57 (1.14, 2.17), 1.08 (0.63–1.85), 0.72 (0.41–1.25), 2.26 (0.98–5.18), 0.47 (0.26–0.86), and 0.85 (95% CI, 0.49–1.46) for Cu, Zn, Se, Mn, Co, Mo, Sn, Sr., and I, respectively (Table 3). For Sr., adjustment for potential confounding factors did not attenuate the association between Sr. and mortality (Figure 3G), and the multivariate-adjusted HRs and 95% CIs from lowest to highest Sr. categories (Q1, Q2, Q3, and Q4) were 1.00 (reference), 0.71 (95%CI: 0.45–1.14), 0.55 (95%CI: 0.32–0.92), and 0.47 (95%CI: 0.26–0.86), respectively, for all-cause mortality (*P–t* < 0.05). Similarly, compared to the first level, the results for the second, third, and fourth levels of Se were 0.56 (95% CI, 0.34, 0.90), 0.52 (95% CI, 0.32, 0.86), 0.47 (95% CI, 0.28, 0.79), respectively (Figure 3C).

**Table 3:** The association between trace element levels with all-cause mortality.

Trace elements	Cases	Controls	Model 1	Model 2	Model 3
			HR (95% CI)	HR (95% CI)	HR (95% CI)
Copper					
Q1	70	1,292	1.00	1.00	1.00
Q2	82	1,248	**1.21 (0.88–1.67)**	0.96 (0.70–1.33)	0.90 (0.50–1.61)
Q3	104	1,267	**1.55 (1.15–2.10)**	**1.37 (1.00–1.88)**	1.21 (0.69–2.11)
Q4	100	1,249	**1.50 (1.11–2.04)**	**1.62 (1.16–2.25)**	1.61 (0.90–2.87)
P for trend			**0.009**	**0.004**	0.108
Zinc					
Q1	110	1,230	1.00	1.00	1.00
Q2	77	1,282	**1.53 (1.13–2.07)**	1.00 (0.73–1.35)	0.68 (0.49–0.94)
Q3	90	1,273	**2.74 (2.08–3.62)**	1.28 (0.96–1.69)	0.82 (0.60–1.12)
Q4	79	1,270	**3.82 (2.94–4.97)**	**2.18 (1.67–2.85)**	0.81 (0.59–1.12)
P for trend			**<0.001**	**<0.001**	0.073
Selenium					
Q1	144	1,229	1.00	1.00	1.00
Q2	68	1,271	**0.48 (0.36–0.63)**	**0.48 (0.36–0.65)**	**0.56 (0.34–0.90)**
Q3	73	1,259	**0.51 (0.39–0.68)**	**0.52 (0.39–0.69)**	**0.52 (0.32–0.86)**
Q4	71	1,292	**0.48 (0.36–0.63)**	**0.48 (0.36–0.64)**	**0.47 (0.28–0.79)**
P for trend			**<0.001**	**<0.001**	**0.004**
Manganese					
Q1	121	1,228	1.00	1.00	1.00
Q2	86	1,265	**0.71 (0.54–0.94)**	0.82 (0.62–1.09)	1.00 (0.74–1.35)
Q3	65	1,290	**0.55 (0.40–0.74)**	0.75 (0.55–1.02)	0.83 (0.59–1.16)
Q4	84	1,268	**0.71 (0.54–0.94)**	1.12 (0.85–1.49)	**1.57 (1.14–2.17)**
P for trend			**0.004**	0.427	**0.006**
Cobalt					
Q1	86	1,249	1.00	1.00	1.00
Q2	82	1,227	0.99 (0.73–1.34)	0.82 (0.61–1.12)	0.80 (0.47–1.35)
Q3	71	1,254	0.89 (0.65–1.22)	0.87 (0.63–1.19)	0.66 (0.38–1.16)
Q4	81	1,247	1.07 (0.79–1.45)	1.10 (0.81–1.49)	1.08 (0.63–1.85)
P for trend			0.674	0.557	0.769
Molybdenum					
Q1	79	1,238	1.00	1.00	1.00
Q2	75	1,256	**0.92 (0.67–1.26)**	0.72 (0.52–0.98)	0.71 (0.42–1.19)
Q3	84	1,240	**1.03 (0.76–1.40)**	0.79 (0.58–1.07)	0.77 (0.46–1.28)
Q4	82	1,242	**1.01 (0.74–1.38)**	0.89 (0.65–1.22)	0.72 (0.41–1.25)
P for trend			**<0.001**	0.349	0.243
Tin					
Q1	65	1,224	1.00	1.00	1.00
Q2	63	1,353	**1.51 (1.00–2.28)**	1.04 (0.69–1.58)	1.46 (0.62–3.46)
Q3	95	1,206	**2.53 (1.72–3.74)**	1.36 (0.92–2.02)	1.85 (0.80–4.29)
Q4	97	1,193	**3.39 (2.33–4.92)**	**1.51 (1.03–2.21)**	2.26 (0.98–5.18)
P for trend			**<0.001**	**0.035**	0.055
Strontium					
Q1	106	1,218	1.00	1.00	1.00
Q2	93	1,231	0.86 (0.65–1.14)	0.78 (0.59–1.03)	0.71 (0.45–1.14)
Q3	60	1,264	**0.56 (0.41–0.77)**	**0.54 (0.39–0.74)**	**0.55 (0.32–0.92)**
Q4	61	1,263	**0.56 (0.41–0.77)**	**0.71 (0.51–0.97)**	**0.47 (0.26–0.86)**
P for trend			**<0.001**	**0.030**	**0.015**
Iodine					
Q1	72	1,277	1.00	1.00	1.00
Q2	69	1,253	1.39 (0.97–1.99)	1.01 (0.70–1.45)	0.66 (0.35–1.24)
Q3	91	1,236	**1.77 (1.26–2.49)**	1.09 (0.77–1.54)	0.89 (0.51–1.57)
Q4	89	1,218	**2.17 (1.56–3.02)**	1.07 (0.77–1.50)	0.85 (0.49–1.46)
P for trend			**<0.001**	0.349	0.551

Model 1: unadjusted model.

Model 2: adjusted for age and sex.

Model 3: adjusted for age, sex, education level, race, marital status, tobacco smoking, alcohol drinking, BMI, PIR category, and physical activity.

Q, quartile. PIR, poverty-income ratio. BMI, body mass index. HR, Hazard ratio. CI, confidence interval.

Besides, Cox regression models with restricted cubic splines showed statistically significant non-linear associations for Mn and all-cause mortality (*P* for non-linearity <0.05, Figure 3D), and the multivariate-adjusted HRs and 95% CIs from lowest to highest Mn categories (Q1, Q2, Q3, and Q4) were 1.00 (reference), 1.00 (95%CI: 0.74–1.35), 0.83 (95%CI: 0.59–1.16), and 1.57 (95%CI: 1.14–2.17), respectively, for all-cause mortality (*P–t* < 0.05). Similarly, there was also a non-linear association between cobalt, zinc and all-cause mortality (Figures 3B,E). The non-linear association of other trace elements with mortality is not observed (Figures 3A,F,H,I).

### 3.3. Overall effects of mixed trace elements on all-cause mortality risk

**Figure 4:**
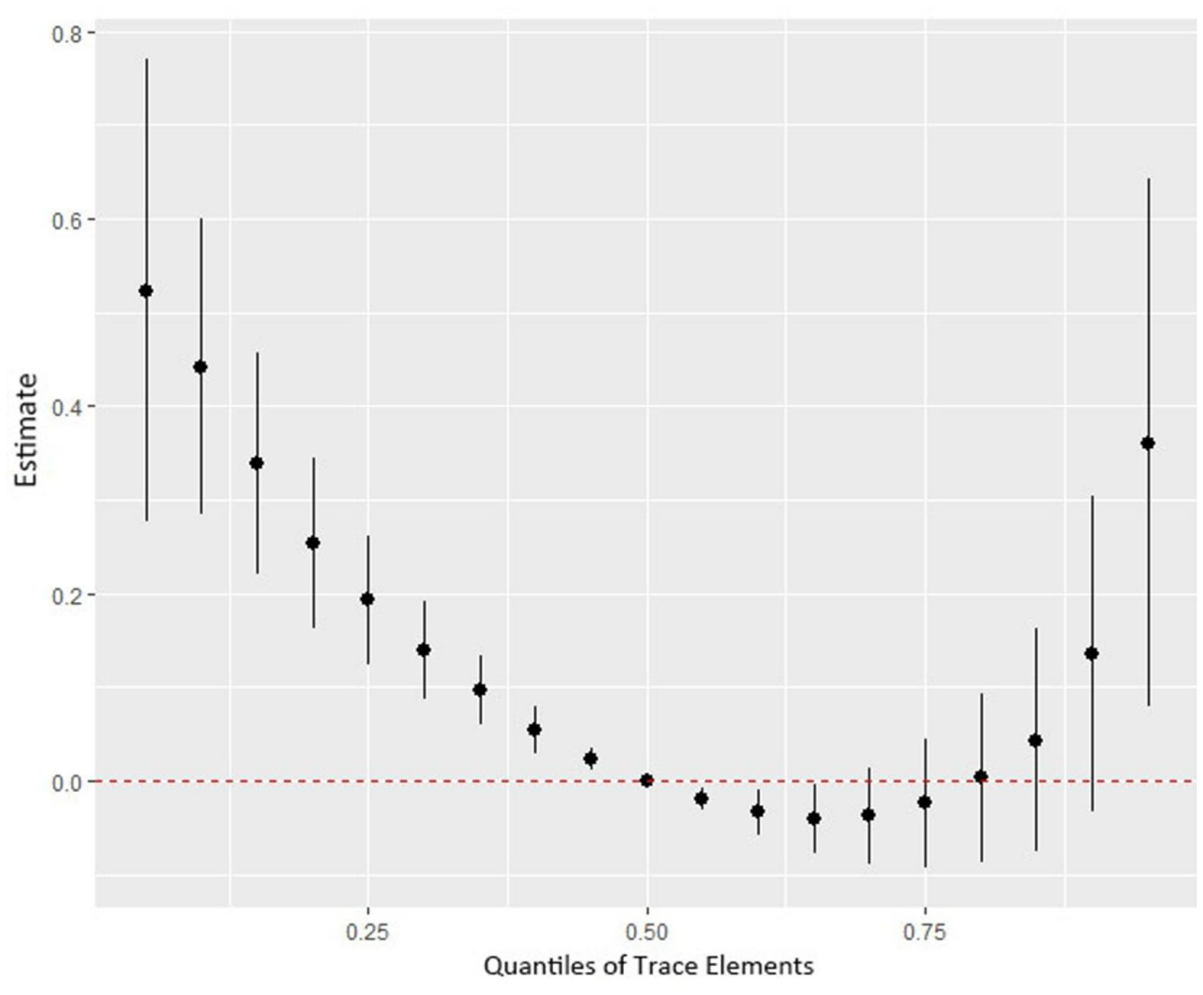
The joint effect (95% CI) of the trace elements mixture on mortality risk was estimated by the BKMR model when all the trace elements at particular percentiles were compared to all trace elements at their 50th percentile. The results were adjusted for age, sex, education level, race/ethnicity, marital status, tobacco smoking, alcohol drinking, BMI, PIR category, and physical activity. BKMR: Bayesian Kernel Machine Regression.

**Figure 5:**
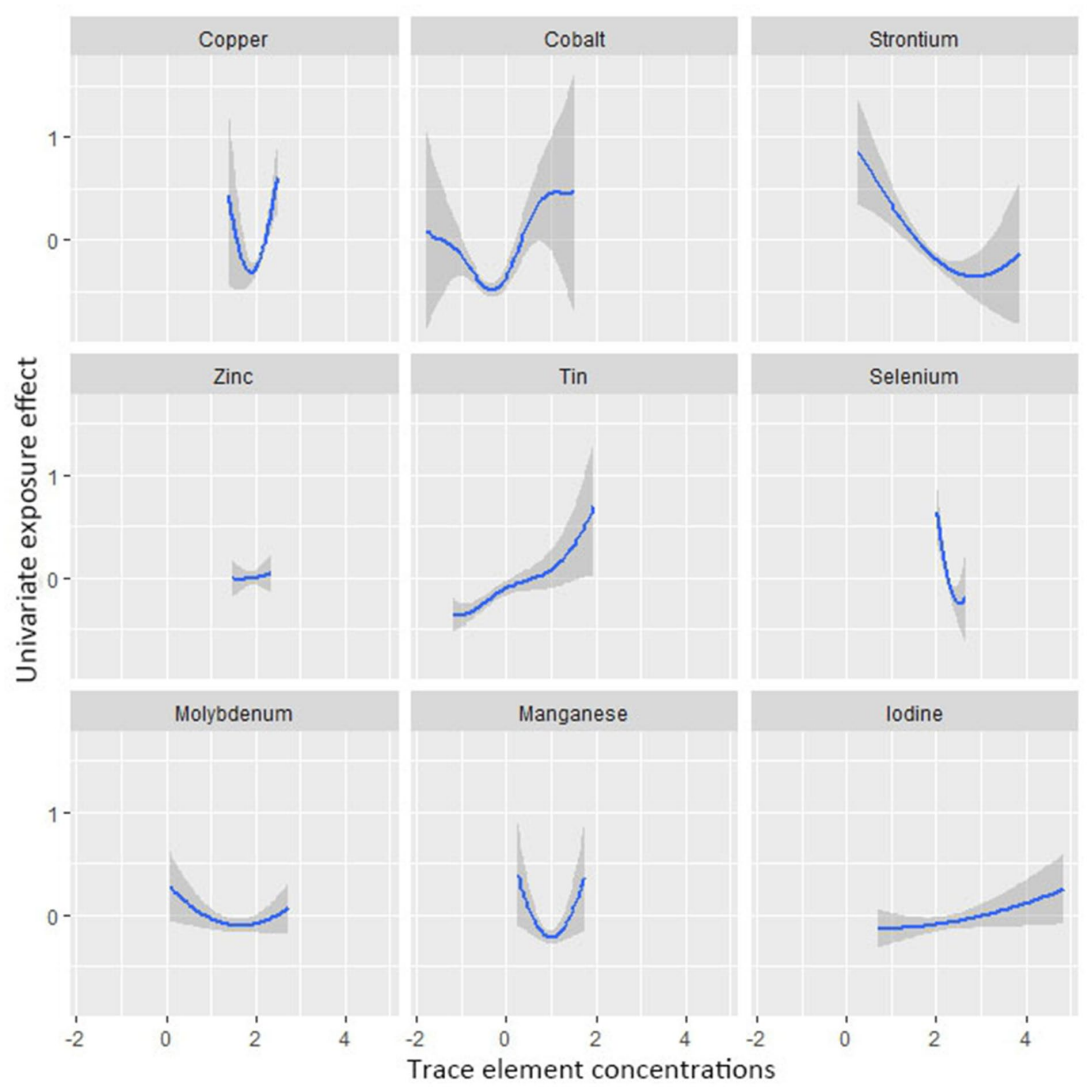
Univariate exposure-response functions and 95% confidence interval between trace element concentrations and all-cause mortality while fixing the concentrations of other trace elements at median values.

**Figure 6:**
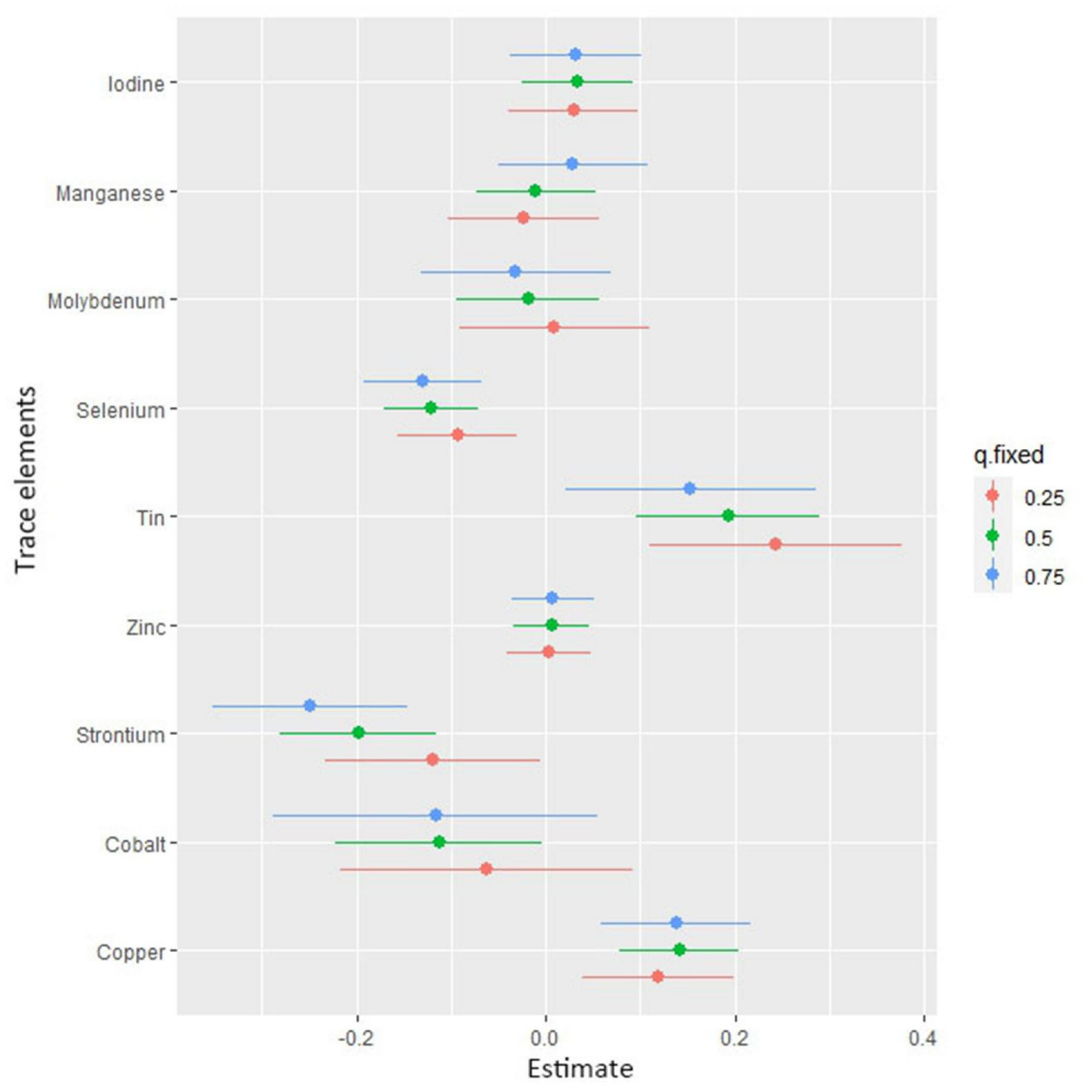
Associations of single trace element with mortality risk. The plot shows the change in risks of all-cause mortality with a 95% confidence interval in a single trace element when all other trace elements were held at their corresponding 25th (red), 50th (green), or 75th (blue) percentile, respectively.

Figure 4 shows the combined association with the risk of mortality when all trace elements exposures were kept at a certain percentile compared to all exposures maintained at the median value. A significant negative correlation was observed between mixture exposure and mortality when the mixture was at the 60th percentile or lower. The posterior inclusion probabilities (PIPs) for the BKMR model are shown in Supplementary Table S2, with the largest PIPs for selenium, tin, and strontium. Tin showed a significant positive trend with the highest posterior inclusion probability (PIP = 0.999). Meanwhile, Figure 5 shows the association of each trace element with all-cause mortality when the other trace elements were taken as median values. When the other trace elements were fixed at the median, Sn was positively associated with the risk of mortality, while Se and Sr. were negatively correlated with the risk of mortality. Furthermore, we also found that Sn displayed a significant and positive effect on the risk of all-cause mortality, and Se and Sr. were negatively associated with all-cause mortality when other trace elements were fixed at the 25th, 50th, and 75th percentiles (Figure 6).

## 4. Discussion

Along with the increasing socio-economic level and the improvement of life quality, the awareness of trace elements in the prevention and treatment of disease is constantly improving, thus adequate attention and effective management of trace element exposure are highly necessary. To our knowledge, few studies assessed the relationship between multiple trace elements and all-cause mortality. Our study systematically examined the independent and joint association between Cu, Zn, Se, Mn, Co, Mo, Sn, Sr, and I with all-cause mortality risk in a large prospective study based on a nationally representative sample of U.S. individuals. Our results showed: (a) manganese may increase all-cause mortality risk; (b) higher selenium and strontium concentrations were associated with lower all-cause mortality; (c) the non-linear relationship was observed between zinc, cobalt and mortality; (d) the mixture of trace elements was negatively correlated with all-cause mortality.

Trace elements are found in the natural environment and are widely available as one of the factors indispensable for the existence of life forms. Meanwhile, trace elements are extremely common in modern industrial manufacturing and can cause health risks to human beings as a main component of environmental pollutants. Previous studies have shown that deficiency or excess of trace elements can lead to poor pregnancy outcomes and adverse clinical consequences (29, 30). When humans are exposed to excessive trace elements with potential toxicity in various ways, these trace elements can accumulate in human tissues and organs, causing irreversible damage to human health. Copper, for example, extensive evidence suggests that in the general population, excessive copper exposure may be potentially associated with many adverse health outcomes. A systematic review and meta-analysis of 37 studies showed a positive association between copper exposure and increased risk of cardiovascular disease and coronary heart disease (31). A Chinese birth cohort showed that high Cu levels were associated with a higher risk of preterm, early-term, and late- or post-term birth (32). However, the dose–response relationship between serum copper and all-cause mortality was not observed in our study.

While trace element exposure exceeding the human acceptable limit can result in health hazards, some elements are also essential trace elements for maintaining some of the body’s functions. Taking selenium as an example, selenium is one of the essential trace elements. If selenium is deficient within the body, the cells of the body will lack the ability to defend themselves and become less resistant to infection (33). However, current studies regarding selenium and mortality risk are still controversial. A meta-analysis including 12 cohort studies and 2 case–control studies showed that lower circulating selenium concentrations were significantly associated with a higher risk of all-cause mortality and CVD mortality in the general population (34). A prospective cohort study of 688 elderly Swedes found that serum selenium concentrations in the lowest quartile increased the risk of all-cause mortality by 43% and the risk of CVD mortality by 56% compared to the highest quartile (35). Our findings also show that selenium reduces the risk of all-cause mortality. A single-center, double-blind randomized controlled trial study, however, revealed that taking a 300 μg daily dose of selenium supplements for 5 years may increase the risk of all-cause mortality after 10 years (36). Another meta-analysis of 43 randomized controlled studies showed no significant association between selenium supplementation alone and all-cause mortality (37). Our results also show that high levels of urinary strontium are associated with lower all-cause mortality.

We also observed non-linear associations between cobalt, zinc and all-cause mortality, with protective effects at lower doses and health hazards associated with high-dose intake. Our findings also suggest that relatively higher concentrations of manganese in the blood may increase the risk of all-cause mortality. However, studies on the association of these trace elements with the risk of all-cause mortality are rare. Numerous studies have been carried out to describe the neurotoxic mechanisms of manganese (38, 39). Further studies are needed to explore the association between the dose–response relationship of these trace elements in the body and the risk of all-cause mortality and other diseases.

More importantly, based on the analysis of the relationship between nine individual trace elements and all-cause mortality, our study used the BKMR model to estimate the impact of trace elements mixed exposure on mortality. Our studies have observed a negative association between mixed trace element exposure and all-cause mortality. However, the actual exposure in the population is characterized by multiple sources, diversity, and simultaneity. Exposure assessments for single elements are often focused and significant risk assessments associated with joint exposures are often ignored, so health risk assessments for mixed exposures also need to be considered in epidemiological studies. In addition, there is the problem that existing research remains difficult to explain the underlying mechanisms of mixing trace elements with health and larger population-based real-world studies and *in vivo* experiments are needed to confirm the effects of mixing exposures.

At present, the rapid development of society and the rapid changes in human lifestyles are leading to increasingly frequent exposures to environmental pollution (including metals and metalloids), and this increases the risk of premature mortality. And this phenomenon will be even more pronounced in the susceptible population. In the future, further attention should be paid to the general and susceptible populations and their associated disease burden, as well as to identify associated health risk behaviors in the related populations.

Therefore, conducting effective biological monitoring in real-world exposure scenarios, exploring the association between long-term low-level trace elements and mortality risk, and validating the findings in large-scale multicenter, population-based prospective studies may be the future direction of environmental and nutritional epidemiology. Moreover, blood and urine are often used as biological carriers to reflect trace element exposure levels in the body. We also need attention because trace elements in bodily fluids may be regulated by stress or inflammation and may not simply reflect nutritional intake or environmental exposure. Hence the observed increases or decreases in trace element levels in studies should be interpreted with caution. For the different trace elements, biological monitoring in different biospecimens (blood, urine, hair, nails, etc.) should also be reinforced to explore the best biospecimens to reflect the level of exposure in humans. Previous existing research has proposed that trace elements may cause oxidative damage by inducing reactive oxygen species in the body (6), and may also affect cellular function by altering their structure (and/or concentration) (40), etc., resulting in many adverse health outcomes. Nonetheless, for most trace elements, the underlying regulatory mechanisms are currently unclear. Future long-term, dynamic, multicenter studies in different populations are therefore needed to explore the maximum acceptable threshold concentrations, potential mechanisms, and health implications.

The strengths and limitations of our study deserve comment. The strengths of our research are as follows: firstly, the prospective study design and the large nationally representative sample of the US population were used to investigate the association between concentrations of 9 trace elements and all-cause mortality, and the results are quite well represented. Secondly, the number of deaths obtained from the long-term cohort follow-up provided sufficient strength for our study’s analysis. And thirdly, multiple statistical methods that enable the relationship between individual and trace element mixtures with all-cause mortality to be fully evaluated, and adjustment for relevant socio-economic status, lifestyle factors, and other potential confounders improve the validity of our study findings. Finally, trace element concentrations in the NHANES database were measured using standard methods and subjected to rigorous quality control, ensuring the robustness of our data results.

There are also limitations to our research. Firstly, as an observational study, the relationship between trace elements and all-cause mortality needs to be validated in the future by conducting more large-scale studies and molecular mechanism studies to make the findings more realistic and credible. Secondly, the current study only measured trace element concentrations once, which may bring possible bias for assessing the long-term exposure association intensity. Furthermore, the extrapolation of the present study’s results is limited due to population and other reasons, and more cohort studies are needed in the future to enhance. Thirdly, there were other unmeasured variables in our study (e.g., psychosocial stress or genetic variation) and the existence of unknown confounding effects due to these factors cannot be excluded. Lastly, due to various limitations, this study did not explore the possible interaction pattern (additive, synergistic, antagonistic, etc.) between multiple trace elements.

In summary, a strong correlation between several trace elements *in vivo* and all-cause mortality was observed, suggesting an important role for trace elements in the effective prevention and control of all-cause mortality. Our findings could provide significant public health implications for the prevention of all-cause mortality and generate scientific evidence for the establishment of relevant surveillance standards or guidance on general groups. Future health interventions and strategies should focus on monitoring and managing trace element levels. And conduct further population-based studies and animal experiments to better understand the underlying mechanisms.

## Data availability statement

Publicly available datasets were analyzed in this study. This data can be at: https://www.cdc.gov/nchs/nhanes/Index.htm.

## Author contributions

LS and SZ designed the study and critically revised the manuscript. XY and SW conducted the statistical analysis and wrote the first draft. LS was the guarantor of the manuscript and accepts full responsibility for the work and the conduct of the study, had access to the data, controlled the decision to publish, and attested that all listed authors met authorship criteria and that no others meeting the criteria have been omitted. All authors contributed to the article and approved the submitted version.

## Funding

This work was supported by the National Natural Science Foundation of China (82103912), the China Postdoctoral Science Foundation (2021M700080), and the Shandong Provincial Natural Science Foundation (ZR2020QH302). The funders were not involved in the collection, analysis, or interpretation of data, or the writing or submitting of this report.

## Conflict of interest

The authors declare that the research was conducted in the absence of any commercial or financial relationships that could be construed as a potential conflict of interest.

## Publisher’s note

All claims expressed in this article are solely those of the authors and do not necessarily represent those of their affiliated organizations, or those of the publisher, the editors and the reviewers. Any product that may be evaluated in this article, or claim that may be made by its manufacturer, is not guaranteed or endorsed by the publisher.

## Footnotes

^1^https://www.cdc.gov/nchs/nhanes/index.htm

^2^https://www.cdc.gov/nchs/nhanes/irba98.htm

^3^https://www.cdc.gov/nchs/data/datalinkage

^4^https://www.cdc.gov/nchs/nhis/data-questionnaires-documentation.htm

^5^https://www.R-project.org/

## Glossary

NHANES: The National Health and Nutrition Examination Survey
NCHS: National Center for Health Statistics
CDC: Centers for Disease Control and Prevention
NDI: National death index
BKMR: Bayesian kernel machine regression
95% CI: 95% Confidence interval
HR: Hazard ratio
PIR: Poverty-income ratio
BMI: Body mass index
Co: Cobalt
Mo: Molybdenum
Sr: Strontium
Sn: Tin
Mn: Manganese
Se: Selenium
I: Iodine
Zn: Zinc
Cu: Copper
