# Supplementary material for: Dose–response relationship between multiple trace elements and risk of all-cause mortality: a prospective cohort study
Source: Front Nutr. 2023 Jul 18;10:1205537. doi: 10.3389/fnut.2023.1205537 (PMC10391637; doi:10.3389/fnut.2023.1205537)
Supplement: Supplementary file 1 [file Data_Sheet_1.docx]

Dose-response relationship between multiple trace elements and risk of all-cause mortality: a prospective cohort study

Running title: Trace elements and all-cause mortality

Shaohua Zhao^1,2^  Shaohua Wang^3^  Xiaorong Yang^4,5^ Lin Shen^1,2^*******

1 Department of Geriatric Medicine, Qilu Hospital of Shandong University, Jinan, China
2 Key Laboratory of Cardiovascular Proteomics of Shandong Province, Qilu Hospital of Shandong University, Jinan, China
3 Department of Internal Medicine, Jinan Hospital, Jinan, China
4 Clinical Epidemiology Unit, Qilu Hospital of Shandong University, Jinan, China

5 Clinical Research Center of Shandong University, Qilu Hospital, Cheeloo College of Medicine, Shandong University, Jinan, China

***Correspondence and requests for materials should be addressed to:**

Lin Shen at Department of Geriatric Medicine, Qilu Hospital, Shandong University, 107 Wenhuaxi Road, Jinan, Shandong 250012, China. Tel/Fax: +86-531-82166721; Email: slmm321 @126.com


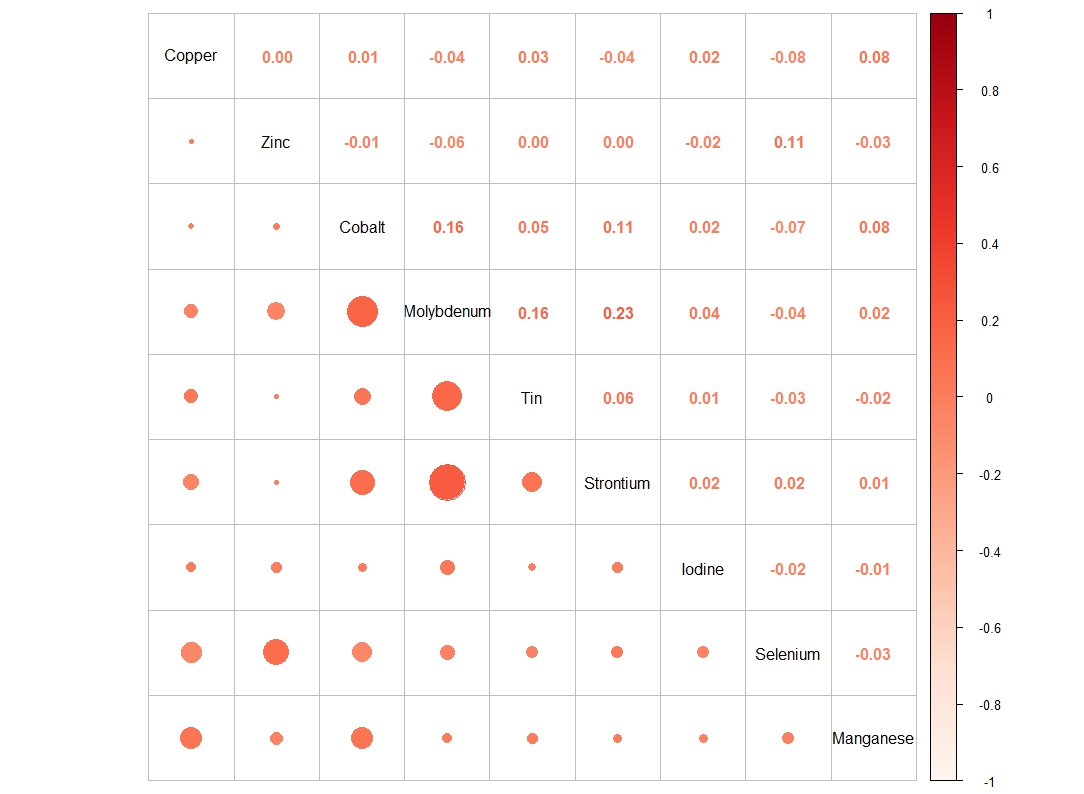


**Figure S1**. Heatmap illustration of correlation for trace elements (manganese, selenium, zinc, copper, cobalt, tin, molybdenum, strontium, iodine).


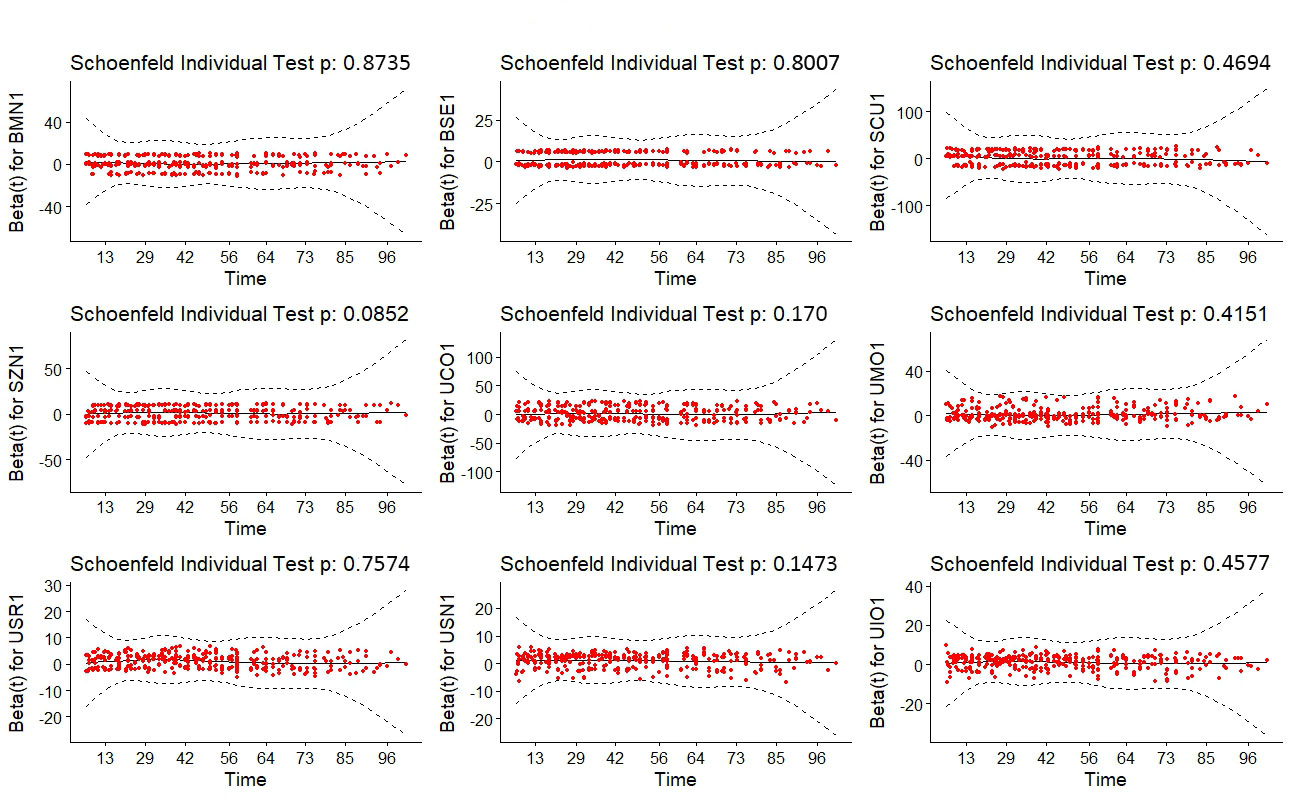


**Figure S2.** The plotted Schoenfeld residuals.

**Table S1.** The lower limit of detection (LLOD) for trace elements (manganese, selenium, zinc, copper, cobalt, tin, molybdenum, strontium, iodine).

| Variable Name | LLOD | Abbreviations |
| --- | --- | --- |
| Copper, Serum | 2.5 µg/dL | Cu |
| Zinc, Serum | 2.9 ug/dL | Zn |
| Manganese, blood | 0.990 µg/L | Mn |
| Selenium, blood | 24.480 µg/L | Se |
| Cobalt, urine | 0.048 µg/L | Co |
| Molybdenum, urine | 0.990 µg/L | Mo |
| Tin, urine | 2.500 µg/L | Sn |
| Strontium, urine | 0.220 µg/L | Sr |
| Iodine - Urine | - | I |

**Table S2.** Posterior inclusion probabilities (PIPs) of the BKMR model.

| Aldehydes | PIP |
| --- | --- |
| Copper, Serum | 0.828 |
| Zinc, Serum | 0.687 |
| Manganese, blood | 0.786 |
| Selenium, blood | 0.999 |
| Cobalt, urine | 0.578 |
| Molybdenum, urine | 0.650 |
| Tin, urine | 0.999 |
| Strontium, urine | 0.999 |
| Iodine - Urine | 0.624 |

Note: adjusted for age, sex, education level, race, marital status, tobacco smoking, alcohol drinking, BMI, PIR category, and physical activity.

**1. Procedures for collecting, storing, and handling specimens.**

Specimen handling conditions, special requirements, and procedures for collection and transport are detailed in the Department of Laboratory Sciences (DLS) Policy and Procedures Manual. In general, the following matters are included.

i. no fasting or special diet is required prior

ii. Use sterile, lot screened collectors for specimen acquisition

iii. the optimum specimen volume is 1+mL. the minimum volume required is 0.25 mL. a volume of 0.05 mL is measured for one analysis.

iv. Make sure that the sample collection device and container are free of visible contamination ("pre-test") prior to use.

v. Draw blood through a stainless steel needle into a pre-screened vacutainer.

vi. Do not freeze the blood in the collection tube as there is a risk of rupture of the tube. It should be transferred to a pre-screened plastic cryovial before freezing. Transport urine specimens frozen (packed in dry ice during shipment is preferred when possible).

vii. Once received, store the blood collection tubes at refrigerated temperatures (2-8 degrees C). Transfer to pre-screened plastic cryovials prior to freezing. Specimens have been shown to be stable for more than 1 year at ≤ -20°C.

**2. Criteria for specimen rejection. Criteria for unacceptable specimens include.**

i. Contamination. Improper collection procedures, collection equipment or sample handling can contaminate blood through exposure to dust, dirt, etc. Manganese is present in the general environment, is often found in combination with iron and is present in many alloys (especially stainless steel).

ii. Low volume. A minimum volume of 0.25 mL is required. a volume of 0.05 mL is required for one analytical measurement. In all cases, a second blood sample is required.

iii. Specimen characteristics that may compromise test results are indicated above. Reasons for rejection of a sample for analysis include the following (in all cases, request a second urine specimen):

1. Low volume: Method 3018 (15 element): Optimal amount of urine is 1.8+ mL. The volume of urine used for one analysis is 0.5 mL. If only a subset of the elements is required, smaller volumes may be required.

2. Contamination: Improper collection procedures or collection devices can contaminate the urine by contact with dust, dirt, etc.

**3. Procedures for the transfer or referral of specimens, specimen accountability and tracking.**

The location, status and final disposition of specimens will be tracked, at a minimum, through a paper file in the "study folder" (created before the specimen is received by the analyst). In addition to this specimen tracking form, this folder will contain paper printouts of specimen analysis results. Maintain records for at least 3 years. Use only numerical identifiers (e.g. case ID numbers) for specimens within the laboratory to safeguard confidentiality. Access to individual identifiers for specimens will be restricted to the medical director or project coordinator (e.g., non-CDC personnel).

**4. Reporting thresholds**

(1) Reportable range. Elemental concentrations in blood are reportable in the range between the LOD of the method and the maximum calibration value multiplied by the maximum validated additional dilution value. Above the maximum validated concentration, the blood sample is subjected to additional dilution to bring it within the reportable range. rine multi-element values are reportable in the range between the method LOD and the highest calibrator. Above the highest calibrator, extra dilutions are made of the urine sample to bring the concentration within the reportable range. If extra dilution has been necessary, the reported value will exceed the upper end of the reportable range.

(2) Reference ranges (normal values). In this method, 95% of the reference ranges for these elements in the blood are within the range of the calibrator.

(3) Action level. Concentrations observed above the "second upper bound" (defined as "2UB" in the laboratory database) are reported to the QC reviewer as "elevated results". The concentration of 2UB assigned to an element is determined by the study protocol. The protocol for reporting elevated results by the supervisor to the medical staff is determined by the study protocol. Due to the uncertainty of the health implications of elevated concentrations of many of the elements determined with this method, there is no routine notification for elevated levels of every analyte determined with this method. However, it is usually the case that.

**5. Method limits of detection (LODs).**

Method LODs for elements in blood specimens are defined as 3 times s_0_, where s_0_ is an estimate of the standard deviation zero analyte concentration. s0 is the y-intercept of a linear or second-order polynomial regression of standard deviation against concentration (60 measurements of each of the 4 concentration levels of analyte in blood over a time horizon of at least 2 months). The LOD of the method is re-evaluated periodically.

Method limit of quantitation (LOQ): The Division of Laboratory Sciences does not currently utilize limits of quantitation in regards to reporting limits.

QC limits: Quality control limits are calculated based on concentration results obtained in at least 20 separate runs. It is preferable to perform separate analyses on separate days and using multiple calibrator lot numbers, instruments, and analysts to best mimic real-life variability. The statistical calculations are performed using the SAS program developed for the Division of Laboratory Sciences.
